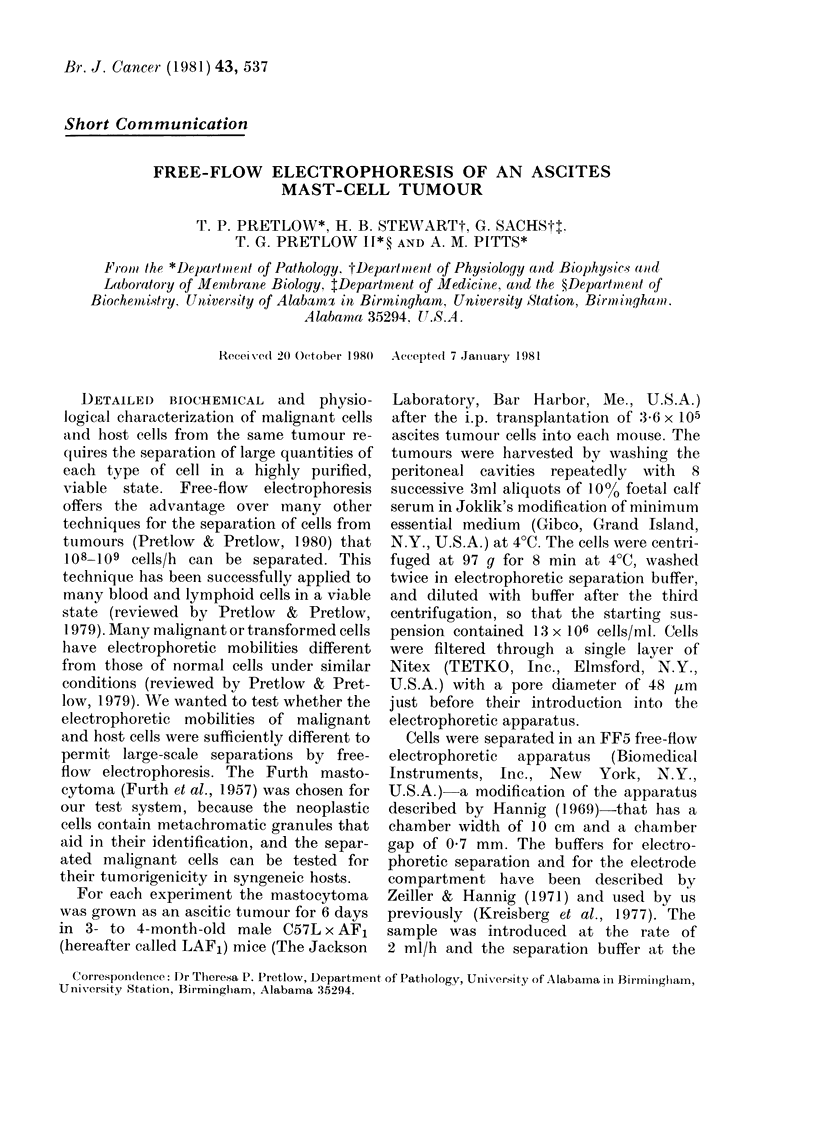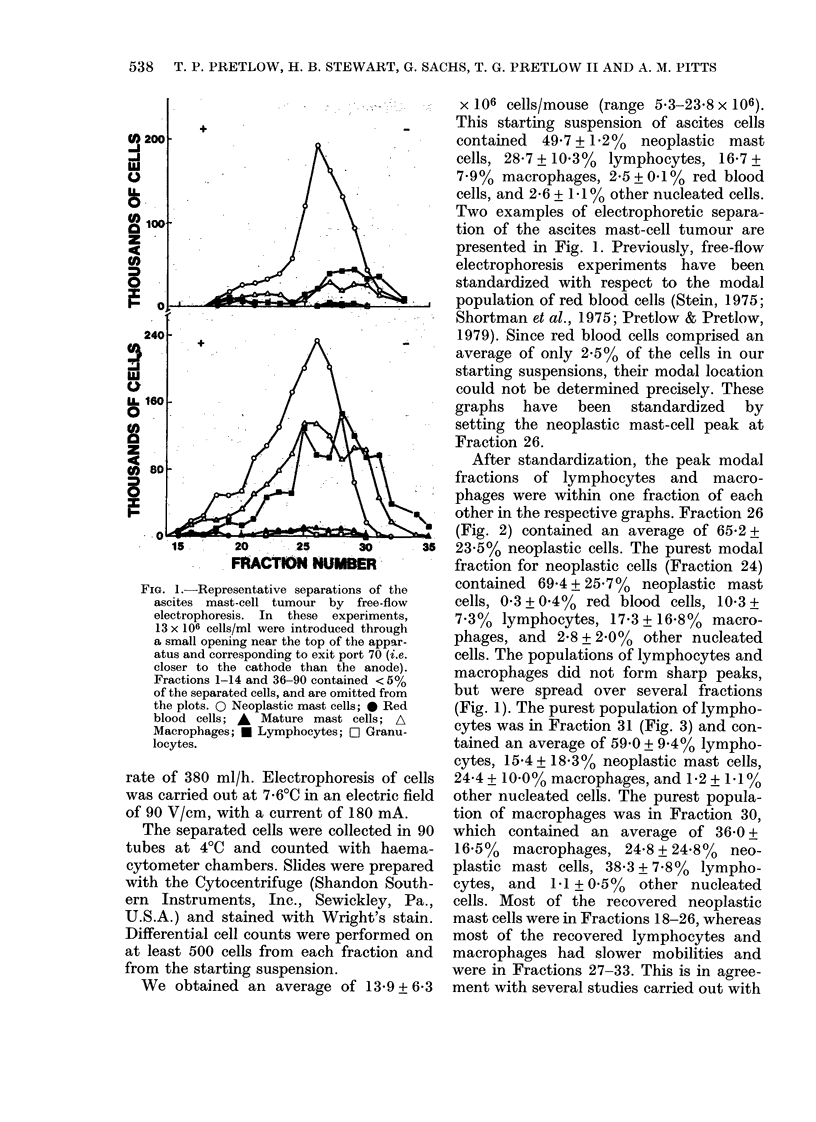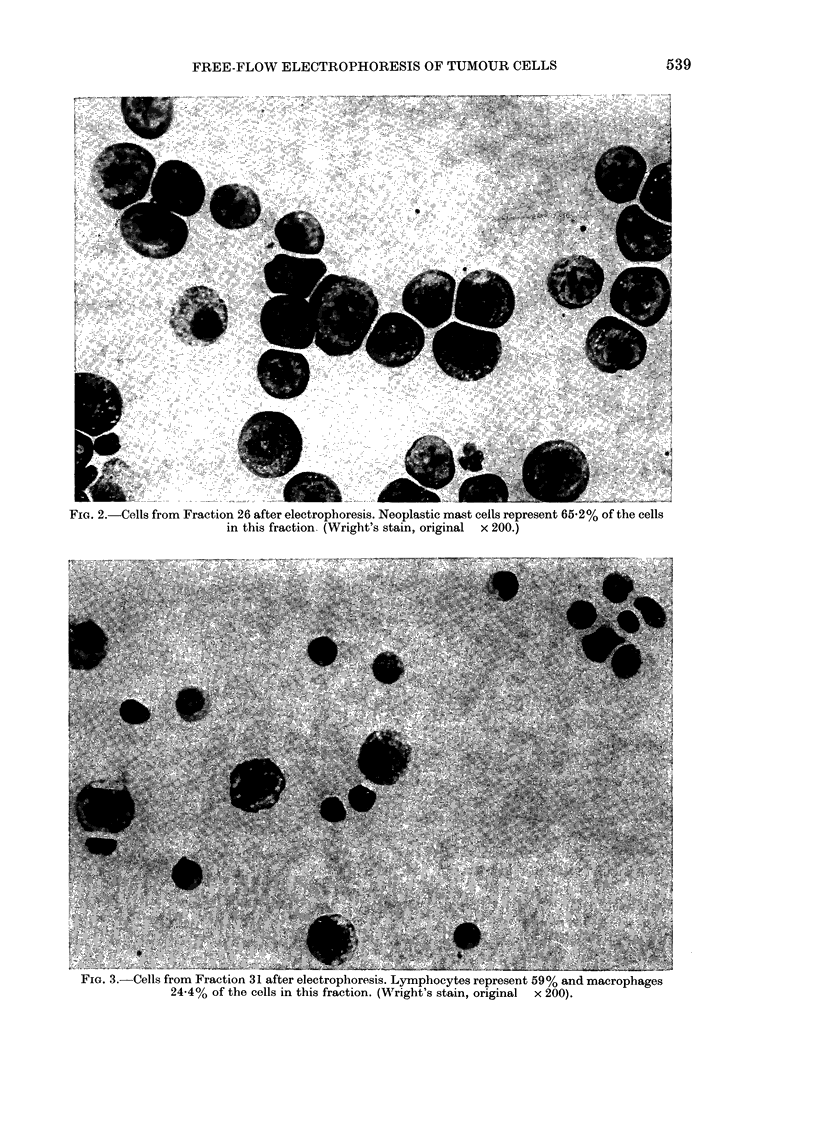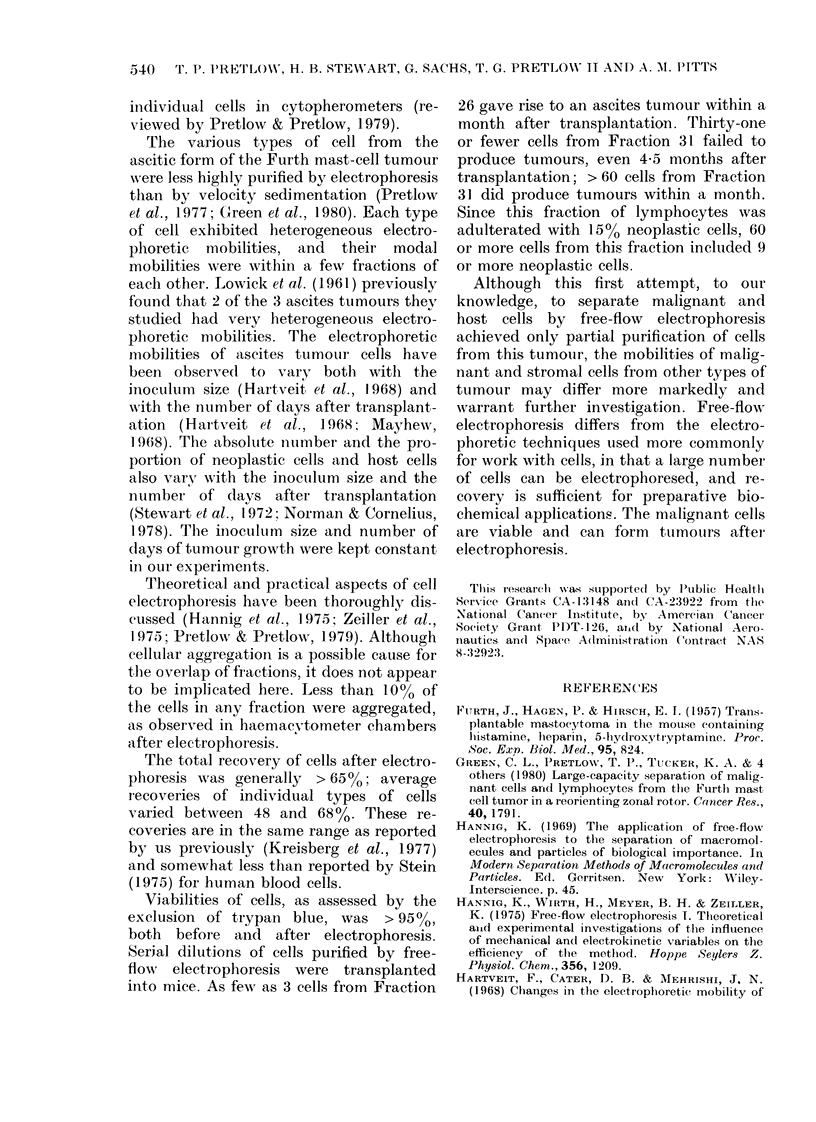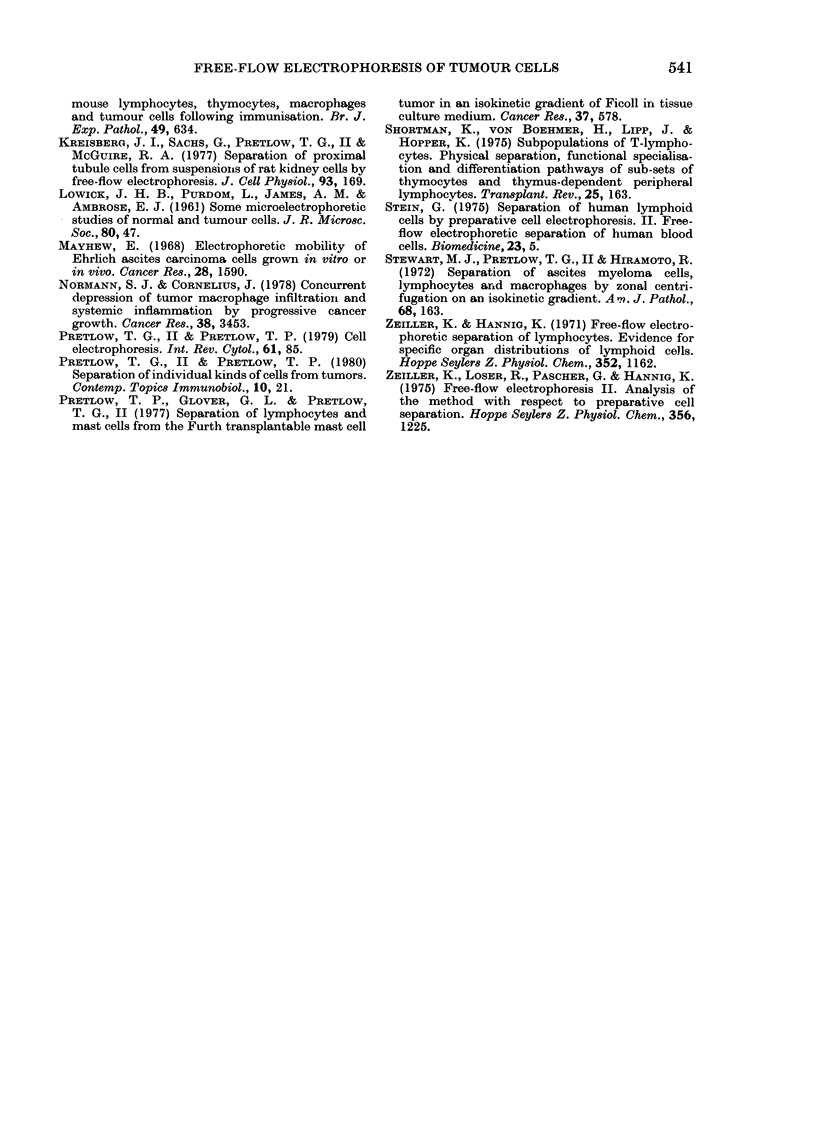# Free-flow electrophoresis of an ascites mast-cell tumour.

**DOI:** 10.1038/bjc.1981.77

**Published:** 1981-04

**Authors:** T. P. Pretlow, H. B. Stewart, G. Sachs, T. G. Pretlow, A. M. Pitts

## Abstract

**Images:**


*Br. J. Cancer* (1981) **43**, 537

***Short Communication***

### FREE-FLOW ELECTROPHORESIS OF AN ASCITES MAST-CELL TUMOUR

T. P. PRETLOW*, H. B. STEWART†, G. SACHS†‡, T. G. PRETLOW II*§ AND A. M. PITTS*

*From the *Department of Pathology, †Department of Physiology and Biophysics and Laboratory of Membrane Biology, ‡Department of Medicine, and the §Department of Biochemistry, University of Alabama in Birmingham, University Station, Birmingham, Alabama 35294, U.S.A.*

Received 20 October 1980 Accepted 7 January 1981

DETAILED BIOCHEMICAL and physiological characterization of malignant cells and host cells from the same tumour requires the separation of large quantities of each type of cell in a highly purified, viable state. Free-flow electrophoresis offers the advantage over many other techniques for the separation of cells from tumours (Pretlow & Pretlow, 1980) that $10^8$–$10^9$ cells/h can be separated. This technique has been successfully applied to many blood and lymphoid cells in a viable state (reviewed by Pretlow & Pretlow, 1979). Many malignant or transformed cells have electrophoretic mobilities different from those of normal cells under similar conditions (reviewed by Pretlow & Pretlow, 1979). We wanted to test whether the electrophoretic mobilities of malignant and host cells were sufficiently different to permit large-scale separations by free-flow electrophoresis. The Furth mastocytoma (Furth *et al.*, 1957) was chosen for our test system, because the neoplastic cells contain metachromatic granules that aid in their identification, and the separated malignant cells can be tested for their tumorigenicity in syngeneic hosts.

For each experiment the mastocytoma was grown as an ascitic tumour for 6 days in 3- to 4-month-old male $C57L \times AF_1$ (hereafter called $LAF_1$) mice (The Jackson Laboratory, Bar Harbor, Me., U.S.A.) after the i.p. transplantation of $3{\cdot}6 \times 10^5$ ascites tumour cells into each mouse. The tumours were harvested by washing the peritoneal cavities repeatedly with 8 successive 3ml aliquots of 10% foetal calf serum in Joklik's modification of minimum essential medium (Gibco, Grand Island, N.Y., U.S.A.) at 4°C. The cells were centrifuged at 97 *g* for 8 min at 4°C, washed twice in electrophoretic separation buffer, and diluted with buffer after the third centrifugation, so that the starting suspension contained $13 \times 10^6$ cells/ml. Cells were filtered through a single layer of Nitex (TETKO, Inc., Elmsford, N.Y., U.S.A.) with a pore diameter of 48 μm just before their introduction into the electrophoretic apparatus.

Cells were separated in an FF5 free-flow electrophoretic apparatus (Biomedical Instruments, Inc., New York, N.Y., U.S.A.)—a modification of the apparatus described by Hannig (1969)—that has a chamber width of 10 cm and a chamber gap of 0·7 mm. The buffers for electrophoretic separation and for the electrode compartment have been described by Zeiller & Hannig (1971) and used by us previously (Kreisberg *et al.*, 1977). The sample was introduced at the rate of 2 ml/h and the separation buffer at the

Correspondence: Dr Theresa P. Pretlow, Department of Pathology, University of Alabama in Birmingham, University Station, Birmingham, Alabama 35294.

FIG. 1.—Representative separations of the ascites mast-cell tumour by free-flow electrophoresis. In these experiments, $13 \times 10^6$ cells/ml were introduced through a small opening near the top of the apparatus and corresponding to exit port 70 (*i.e.* closer to the cathode than the anode). Fractions 1–14 and 36–90 contained $<5\%$ of the separated cells, and are omitted from the plots. ○ Neoplastic mast cells; ● Red blood cells; ▲ Mature mast cells; △ Macrophages; ■ Lymphocytes; □ Granulocytes.

rate of 380 ml/h. Electrophoresis of cells was carried out at 7·6°C in an electric field of 90 V/cm, with a current of 180 mA.

The separated cells were collected in 90 tubes at 4°C and counted with haemacytometer chambers. Slides were prepared with the Cytocentrifuge (Shandon Southern Instruments, Inc., Sewickley, Pa., U.S.A.) and stained with Wright's stain. Differential cell counts were performed on at least 500 cells from each fraction and from the starting suspension.

We obtained an average of $13{\cdot}9 \pm 6{\cdot}3 \times 10^6$ cells/mouse (range $5{\cdot}3$–$23{\cdot}8 \times 10^6$). This starting suspension of ascites cells contained $49{\cdot}7 \pm 1{\cdot}2\%$ neoplastic mast cells, $28{\cdot}7 \pm 10{\cdot}3\%$ lymphocytes, $16{\cdot}7 \pm 7{\cdot}9\%$ macrophages, $2{\cdot}5 \pm 0{\cdot}1\%$ red blood cells, and $2{\cdot}6 \pm 1{\cdot}1\%$ other nucleated cells. Two examples of electrophoretic separation of the ascites mast-cell tumour are presented in Fig. 1. Previously, free-flow electrophoresis experiments have been standardized with respect to the modal population of red blood cells (Stein, 1975; Shortman *et al.*, 1975; Pretlow & Pretlow, 1979). Since red blood cells comprised an average of only 2·5% of the cells in our starting suspensions, their modal location could not be determined precisely. These graphs have been standardized by setting the neoplastic mast-cell peak at Fraction 26.

After standardization, the peak modal fractions of lymphocytes and macrophages were within one fraction of each other in the respective graphs. Fraction 26 (Fig. 2) contained an average of $65{\cdot}2 \pm 23{\cdot}5\%$ neoplastic cells. The purest modal fraction for neoplastic cells (Fraction 24) contained $69{\cdot}4 \pm 25{\cdot}7\%$ neoplastic mast cells, $0{\cdot}3 \pm 0{\cdot}4\%$ red blood cells, $10{\cdot}3 \pm 7{\cdot}3\%$ lymphocytes, $17{\cdot}3 \pm 16{\cdot}8\%$ macrophages, and $2{\cdot}8 \pm 2{\cdot}0\%$ other nucleated cells. The populations of lymphocytes and macrophages did not form sharp peaks, but were spread over several fractions (Fig. 1). The purest population of lymphocytes was in Fraction 31 (Fig. 3) and contained an average of $59{\cdot}0 \pm 9{\cdot}4\%$ lymphocytes, $15{\cdot}4 \pm 18{\cdot}3\%$ neoplastic mast cells, $24{\cdot}4 \pm 10{\cdot}0\%$ macrophages, and $1{\cdot}2 \pm 1{\cdot}1\%$ other nucleated cells. The purest population of macrophages was in Fraction 30, which contained an average of $36{\cdot}0 \pm 16{\cdot}5\%$ macrophages, $24{\cdot}8 \pm 24{\cdot}8\%$ neoplastic mast cells, $38{\cdot}3 \pm 7{\cdot}8\%$ lymphocytes, and $1{\cdot}1 \pm 0{\cdot}5\%$ other nucleated cells. Most of the recovered neoplastic mast cells were in Fractions 18–26, whereas most of the recovered lymphocytes and macrophages had slower mobilities and were in Fractions 27–33. This is in agreement with several studies carried out with

FIG. 2.—Cells from Fraction 26 after electrophoresis. Neoplastic mast cells represent 65·2% of the cells in this fraction. (Wright's stain, original × 200.)

FIG. 3.—Cells from Fraction 31 after electrophoresis. Lymphocytes represent 59% and macrophages 24·4% of the cells in this fraction. (Wright's stain, original × 200).

individual cells in cytopherometers (reviewed by Pretlow & Pretlow, 1979).

The various types of cell from the ascitic form of the Furth mast-cell tumour were less highly purified by electrophoresis than by velocity sedimentation (Pretlow *et al.*, 1977; Green *et al.*, 1980). Each type of cell exhibited heterogeneous electrophoretic mobilities, and their modal mobilities were within a few fractions of each other. Lowick *et al.* (1961) previously found that 2 of the 3 ascites tumours they studied had very heterogeneous electrophoretic mobilities. The electrophoretic mobilities of ascites tumour cells have been observed to vary both with the inoculum size (Hartveit *et al.*, 1968) and with the number of days after transplantation (Hartveit *et al.*, 1968; Mayhew, 1968). The absolute number and the proportion of neoplastic cells and host cells also vary with the inoculum size and the number of days after transplantation (Stewart *et al.*, 1972; Norman & Cornelius, 1978). The inoculum size and number of days of tumour growth were kept constant in our experiments.

Theoretical and practical aspects of cell electrophoresis have been thoroughly discussed (Hannig *et al.*, 1975; Zeiller *et al.*, 1975; Pretlow & Pretlow, 1979). Although cellular aggregation is a possible cause for the overlap of fractions, it does not appear to be implicated here. Less than 10% of the cells in any fraction were aggregated, as observed in haemacytometer chambers after electrophoresis.

The total recovery of cells after electrophoresis was generally >65%; average recoveries of individual types of cells varied between 48 and 68%. These recoveries are in the same range as reported by us previously (Kreisberg *et al.*, 1977) and somewhat less than reported by Stein (1975) for human blood cells.

Viabilities of cells, as assessed by the exclusion of trypan blue, was >95%, both before and after electrophoresis. Serial dilutions of cells purified by free-flow electrophoresis were transplanted into mice. As few as 3 cells from Fraction 26 gave rise to an ascites tumour within a month after transplantation. Thirty-one or fewer cells from Fraction 31 failed to produce tumours, even 4·5 months after transplantation; >60 cells from Fraction 31 did produce tumours within a month. Since this fraction of lymphocytes was adulterated with 15% neoplastic cells, 60 or more cells from this fraction included 9 or more neoplastic cells.

Although this first attempt, to our knowledge, to separate malignant and host cells by free-flow electrophoresis achieved only partial purification of cells from this tumour, the mobilities of malignant and stromal cells from other types of tumour may differ more markedly and warrant further investigation. Free-flow electrophoresis differs from the electrophoretic techniques used more commonly for work with cells, in that a large number of cells can be electrophoresed, and recovery is sufficient for preparative biochemical applications. The malignant cells are viable and can form tumours after electrophoresis.

This research was supported by Public Health Service Grants CA-13148 and CA-23922 from the National Cancer Institute, by Amercian Cancer Society Grant PDT-126, and by National Aeronautics and Space Administration Contract NAS 8-32923.

### REFERENCES

FURTH, J., HAGEN, P. & HIRSCH, E. I. (1957) Transplantable mastocytoma in the mouse containing histamine, heparin, 5-hydroxytryptamine. *Proc. Soc. Exp. Biol. Med.*, **95**, 824.

GREEN, C. L., PRETLOW, T. P., TUCKER, K. A. & 4 others (1980) Large-capacity separation of malignant cells and lymphocytes from the Furth mast cell tumor in a reorienting zonal rotor. *Cancer Res.*, **40**, 1791.

HANNIG, K. (1969) The application of free-flow electrophoresis to the separation of macromolecules and particles of biological importance. In *Modern Separation Methods of Macromolecules and Particles*. Ed. Gerritsen. New York: Wiley-Interscience. p. 45.

HANNIG, K., WIRTH, H., MEYER, B. H. & ZEILLER, K. (1975) Free-flow electrophoresis I. Theoretical and experimental investigations of the influence of mechanical and electrokinetic variables on the efficiency of the method. *Hoppe Seylers Z. Physiol. Chem.*, **356**, 1209.

HARTVEIT, F., CATER, D. B. & MEHRISHI, J. N. (1968) Changes in the electrophoretic mobility of mouse lymphocytes, thymocytes, macrophages and tumour cells following immunisation. *Br. J. Exp. Pathol.*, **49**, 634.

KREISBERG, J. I., SACHS, G., PRETLOW, T. G., II & McGUIRE, R. A. (1977) Separation of proximal tubule cells from suspensions of rat kidney cells by free-flow electrophoresis. *J. Cell Physiol.*, **93**, 169.

LOWICK, J. H. B., PURDOM, L., JAMES, A. M. & AMBROSE, E. J. (1961) Some microelectrophoretic studies of normal and tumour cells. *J. R. Microsc. Soc.*, **80**, 47.

MAYHEW, E. (1968) Electrophoretic mobility of Ehrlich ascites carcinoma cells grown *in vitro* or *in vivo*. *Cancer Res.*, **28**, 1590.

NORMANN, S. J. & CORNELIUS, J. (1978) Concurrent depression of tumor macrophage infiltration and systemic inflammation by progressive cancer growth. *Cancer Res.*, **38**, 3453.

PRETLOW, T. G., II & PRETLOW, T. P. (1979) Cell electrophoresis. *Int. Rev. Cytol.*, **61**, 85.

PRETLOW, T. G., II & PRETLOW, T. P. (1980) Separation of individual kinds of cells from tumors. *Contemp. Topics Immunobiol.*, **10**, 21.

PRETLOW, T. P., GLOVER, G. L. & PRETLOW, T. G., II (1977) Separation of lymphocytes and mast cells from the Furth transplantable mast cell tumor in an isokinetic gradient of Ficoll in tissue culture medium. *Cancer Res.*, **37**, 578.

SHORTMAN, K., VON BOEHMER, H., LIPP, J. & HOPPER, K. (1975) Subpopulations of T-lymphocytes. Physical separation, functional specialisation and differentiation pathways of sub-sets of thymocytes and thymus-dependent peripheral lymphocytes. *Transplant. Rev.*, **25**, 163.

STEIN, G. (1975) Separation of human lymphoid cells by preparative cell electrophoresis. II. Free-flow electrophoretic separation of human blood cells. *Biomedicine*, **23**, 5.

STEWART, M. J., PRETLOW, T. G., II & HIRAMOTO, R. (1972) Separation of ascites myeloma cells, lymphocytes and macrophages by zonal centrifugation on an isokinetic gradient. *Am. J. Pathol.*, **68**, 163.

ZEILLER, K. & HANNIG, K. (1971) Free-flow electrophoretic separation of lymphocytes. Evidence for specific organ distributions of lymphoid cells. *Hoppe Seylers Z. Physiol. Chem.*, **352**, 1162.

ZEILLER, K., LOSER, R., PASCHER, G. & HANNIG, K. (1975) Free-flow electrophoresis II. Analysis of the method with respect to preparative cell separation. *Hoppe Seylers Z. Physiol. Chem.*, **356**, 1225.